# Farmland pest recognition based on Cascade RCNN Combined with Swin-Transformer

**DOI:** 10.1371/journal.pone.0304284

**Published:** 2024-06-06

**Authors:** Ruikang Xu, Jiajun Yu, Lening Ai, Haojie Yu, Zining Wei

**Affiliations:** 1 Mechanical and Electrical Engineering Department, Qingdao University of Technology, Linyi, Shandong, China; 2 Management Engineering Department, Qingdao University of Technology, Linyi, Shandong, China; Suez Canal University, EGYPT

## Abstract

Agricultural pests and diseases pose major losses to agricultural productivity, leading to significant economic losses and food safety risks. However, accurately identifying and controlling these pests is still very challenging due to the scarcity of labeling data for agricultural pests and the wide variety of pest species with different morphologies. To this end, we propose a two-stage target detection method that combines Cascade RCNN and Swin Transformer models. To address the scarcity of labeled data, we employ random cut-and-paste and traditional online enhancement techniques to expand the pest dataset and use Swin Transformer for basic feature extraction. Subsequently, we designed the SCF-FPN module to enhance the basic features to extract richer pest features. Specifically, the SCF component provides a self-attentive mechanism with a flexible sliding window to enable adaptive feature extraction based on different pest features. Meanwhile, the feature pyramid network (FPN) enriches multiple levels of features and enhances the discriminative ability of the whole network. Finally, to further improve our detection results, we incorporated non-maximum suppression (Soft NMS) and Cascade R-CNN’s cascade structure into the optimization process to ensure more accurate and reliable prediction results. In a detection task involving 28 pest species, our algorithm achieves 92.5%, 91.8%, and 93.7% precision in terms of accuracy, recall, and mean average precision (mAP), respectively, which is an improvement of 12.1%, 5.4%, and 7.6% compared to the original baseline model. The results demonstrate that our method can accurately identify and localize farmland pests, which can help improve farmland’s ecological environment.

## 1 Introduction

Agricultural pests and diseases are a critical concern, precipitating substantial reductions in crop yields and potentially complete losses, thus significantly impacting the economic welfare of farmers and the stability of global food supplies and posing dire threats to food safety and quality via contamination [1, 2]. The extensive deployment of pesticides as a control measure escalates production costs and contributes to environmental pollution while disrupting ecological equilibriums, affecting beneficial organisms, and diminishing biodiversity. This scenario further involves the social and economic sustainability of agriculture-dependent nations. It introduces latent threats to environmental security, encompassing the health of water resources and soil integrity [3].

Traditional pest and disease monitoring and management relies heavily on manual observation and statistical methods; however, this approach usually faces the problem that pests and diseases often have already broken out within their range, making it challenging to take timely pest and disease control measures [4, 5]. In addition, the identification of some pests and diseases often requires a high degree of expert knowledge, for less experienced farmers are not able to accurately find and judge the types of pests and diseases, which may result in the abuse and misuse of pesticides, as well as pesticide residues in crops, affecting the ecological environment of the farmland. Due to the diversity and complexity of crop pests, simple manual detection methods have made it challenging to meet the needs of modern agricultural science and technology development.

In recent years, the rapid development of deep learning technology has provided new ways to address these challenges. The application of deep learning methods to the intelligent recognition of crop pest images has become an important research direction, providing a potential solution to the limitations of traditional pest management methods. For example, Sourav et al. [6] pioneered an intelligent model leveraging transfer learning (TL) and a deep convolutional neural network (DCNN) tailored explicitly for identifying jute pests. This model demonstrated remarkable proficiency, achieving a final accuracy of 95.86% across four major jute pest categories. Concurrently, Chu et al. [7] developed a sophisticated multi-scale granary pest identification model. This model, employing the advanced YOLOv5 target detection algorithm complemented with BiFPN, DIOU-NMS, and ECA modules, excelled in identifying seven common granary pests, achieving an impressive average accuracy of 98.2%. Further contributions in this field were made by Jia et al. [8], who utilized transfer learning and attention mechanisms to construct and refine VGG, ResNet, and MobileNet recognition models. This integrative approach yielded a notable average recognition accuracy of 93.65% across 14 pest categories in pest data sets. Additionally, the innovative MPest-RCNN, as proposed by Wang et al. [9], represents a significant advancement in this domain. This method offers a precise and efficient solution for identifying and enumerating apple pests by harnessing deep learning and data reorganization. In practical applications, their experimental accuracy on three typical pests in apple orchards was recorded at an exceptional 99.11%.

However, there are some limitations to the above methods. Existing models can only handle specific datasets, which makes it difficult to deal well with a wide range of pests and requires a large amount of labeled data for support [10–12]. However, these limitations become more significant in the context of scarcity of labeled data on farmland pests and considering the wide variety of pest species, their different morphologies, and the often dense occurrence of the pests. We propose a new two-stage target detection method that combines the Cascade R-CNN [13] and Swin Transformer [14] models to address these issues. First, we employed random cut-and-paste and traditional online enhancement methods to extend the dataset to address the limited labeled pest samples challenge. To address the problem of diverse pest species and morphology, we introduced the SCF-FPN layer after the backbone network. This layer integrates the SCF self-attention mechanism to enhance the basic image features by calculating the correlation within the sliding window size and learning to extract important local area features from it. Secondly, feature fusion blocks fuse contextual information and enhance feature representation. In addition, it works synergistically with a feature pyramid network (FPN) to better capture targets of different sizes, including oversized, medium, and small pests. Since pests are often densely packed together, traditional NMS may discard some practical bounding boxes, so we introduce Soft NMS [15] to preserve these bounding boxes somewhat and improve the detection. Finally, we utilize the cascade structure of Cascade R-CNN to filter out the false detection samples at each stage. The results show that our method achieves good detection results in the detection task of 28 pest species.

Our contributions:

- We propose a new pest detection framework that performs well even with many pest categories and limited labeled images.
- We augment the data for a small number of labeled and unbalanced category samples to prevent undesirable effects.
- We design a self-attentive module SCF with a flexible sliding window, which can enhance the neighborhood features by changing the sliding window size of feature maps of different sizes and combining with FPN to enhance the capture of large, medium, and small targets.

## 2 Related work

### Cascade R-CNN

Compared to traditional single-stage target detection models [16–18], Cascade R-CNN introduces a cascade structure that enhances the model’s accuracy through a multi-stage detection process. At the heart of this structure lies the Cascade Detector, comprising a series of cascaded detectors. Each detector operates as an independent target detection model, taking candidate frames generated by the Region Proposal Network (RPN) [19] and progressively filtering out the final target frames.

During the multi-stage training process, the cascade detectors selectively utilize a subset of candidate frames as inputs for the subsequent stages. This design enables each cascade stage to recapture local details and correlate cascade structure, bringing the advantage of reducing false detections while improving the recall rate through iterative screening in multiple stages. Additionally, each cascade stage focuses on different target features and difficulty stages, enhancing detection accuracy. The excellent performance of Cascade R-CNN in object detection makes it a versatile solution for various object detection tasks. In agricultural settings, for instance, the model has been adapted to perform highly accurate object detection tasks. These include identifying and classifying various agricultural pests, monitoring crop health, and facilitating more efficient and sustainable farming practices [20, 21]. Similarly, in industrial contexts, Cascade R-CNN’s enhanced detection capabilities have been pivotal in automating quality control processes, detecting manufacturing defects, and ensuring safety in automated systems [22, 23].

### Swin Transformer

Swin Transformer is a deep learning model based on the Transformer architecture [24], which has achieved significant performance gains in image processing tasks. Its architecture comprises two key components: local window interaction and global feature integration. The local window interaction module splits the input image into a series of overlapping local windows and operates the Transformer’s self-attention mechanism within each window. This local window interaction helps the model capture local details and correlate information in the image. Second, the global feature integration module integrates the outputs of the local window interaction module to obtain global contextual information. This module enables the model to better understand the semantic information of the whole image by fusing and reorganizing features from different windows. Compared to traditional convolutional networks, the Swin Transformer reduces computational and memory requirements by using localized window interactions to partition the image into multiple smaller chunks for processing. This allows Swin Transformer to process larger-sized images while keeping the computational cost low. Its Local Window Interaction Module can adaptively process images of different sizes without adjusting the model’s parameters. This makes the Swin Transformer very scalable in processing images of different sizes. As a result, many variants of swim-transform have been born, showing excellent versatility and effectiveness in target detection, fine-grained recognition, medical segmentation, action recognition, and so on [25–27].

### Attention mechanism

In recent years, attention mechanisms have developed rapidly in artificial intelligence, especially in natural language processing (NLP) and computer vision (CV) [28–30]. These mechanisms enable models to focus on specific input parts, leading to more accurate context-aware processing. It allows the model to capture the relationships between different elements in the input by assigning attention weights to each component based on its relevance to other elements. This allows the model to model long-range dependencies and effectively capture fine-grained dependencies between elements. In the field of NLP BERT, GPT and other models based on the Transformer architecture have made breakthroughs in various language understanding tasks by utilizing the attention mechanism to improve the understanding of text context [31, 32]. In computer vision, the attention mechanism has been used for multiple tasks such as image classification, target detection, image generation, etc. Vision Transformer (ViT) [33] is a landmark model that applies Transformer to image processing by slicing the image into sequences and utilizing the self-attention mechanism to deal with these sequences, thus effectively improving the model’s ability to capture image features. In the multimodal domain, OpenAI’s DALL-E and CLIP models [34, 35]demonstrate excellent capabilities in understanding visual and textual content.

However, traditional methods usually rely on neighborhood correlation when computing image autocorrelation [36, 37]. In contrast, the SCF module proposed in this paper eliminates redundant parameters by capturing the dependencies between neighboring elements in the input through a sliding window and realizing contextual feature awareness through simple connections.

Considering the inherent difficulty in distinguishing insect images, we compare the performance of several commonly used target detection algorithms, as summarized in Table 1. These algorithms include Mask R-CNN [38], Faster R-CNN [19], RetinaNet [39], and Cascade R-CNN [13]. By evaluating the ImageNet-1K dataset [40], Table 1 reveals that the architecture combining Cascade R-CNN with Swin Transformer achieves the best detection speed (fps), box AP, and mask AP. However, it is worth noting that this architecture exhibits a larger model size compared to R-50-FPN and R-101-FPN [41], although it remains smaller than the backbone network architecture of Cascade R-CNN modeled as X-101–64x4d-FPN [42].

**Table 1:** Performance comparison of different detection models.

Method	Backbone	Mem (GB)	Inf time (fps)	box AP	mask AP
Mask R-CNN	R-50-FPN	4.4	16.1	38.2	34.7
	R-101-FPN	6.4	13.5	40.0	36.1
	X-101-64x4d-FPN	10.7	8.0	42.8	38.4
Faster_rcnn	R-50-FPN	4.0	21.1	37.4	-
	R-101-FPN	6.0	15.6	39.4	-
	X-101-64x4d-FPN	10.3	9.4	42.1	-
RetinaNet	R-50-FPN	**3.8**	19.0	36.5	-
	R-101-FPN	5.7	15.0	38.5	-
	X-101-64x4d-FPN	10.0	8.7	41.0	-
Cascade R-CNN	R-50-FPN	6.0	11.2	41.2	35.9
	R-101-FPN	7.9	9.8	42.9	37.3
	X-101-64x4d-FPN	12.2	6.7	45.3	39.2
**Ours**	Swin-S-FPN	11.9	**6.1**	**52.6**	**46.2**

## 3 Our approach

**Fig 1:**
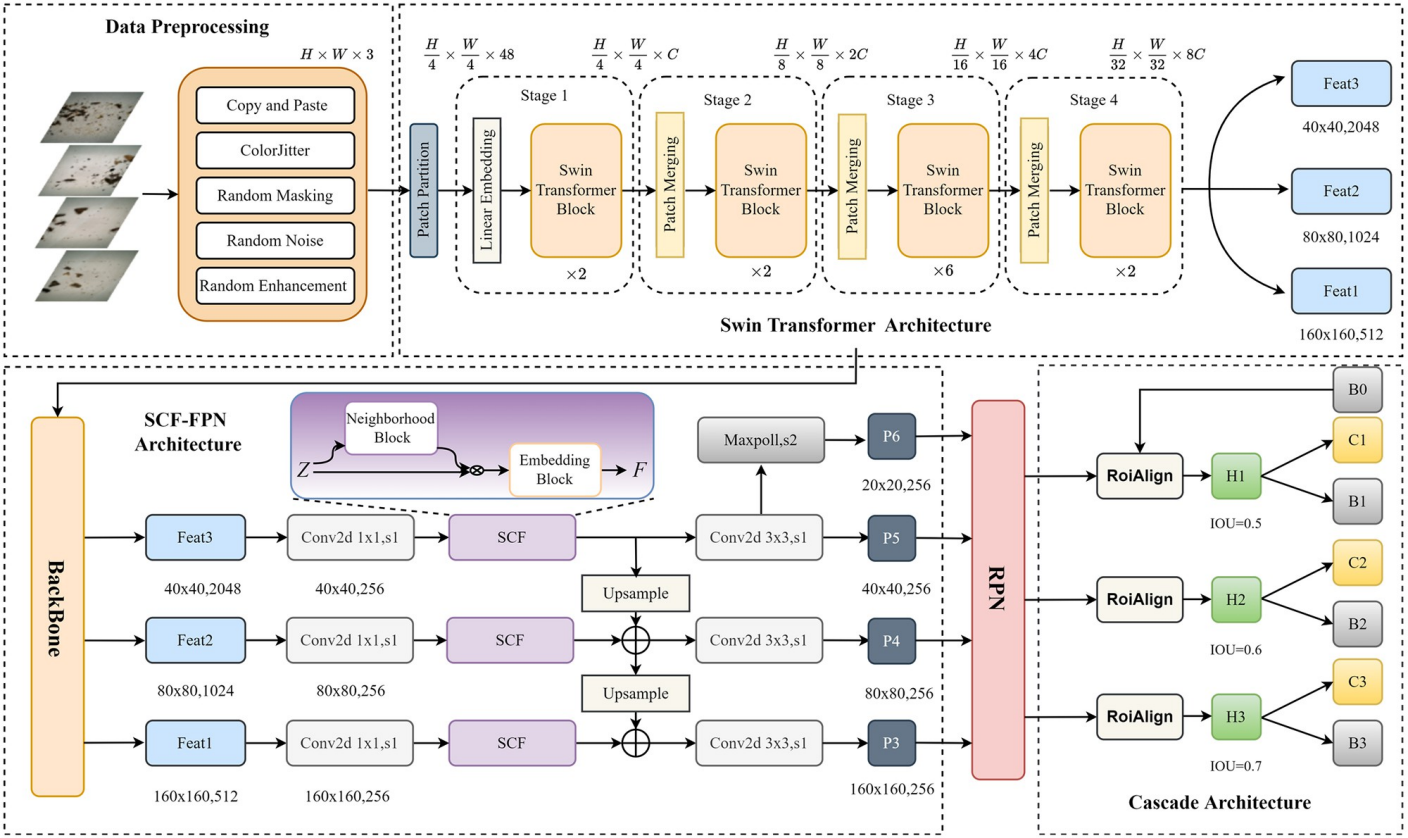
The overall framework of the pest detection network is based on the combination of Cascade RCNN and Swin Transformer. We feed the data-expanded image into the Swin Transformer to extract the base features. These features, namely feat1=(160,160,256), feat2=(80,80,512), and feat3=(40,40,1024), correspond to small, medium, and large targets, respectively. Subsequently, we utilize the SCF-FPN to enhance these base features. The resulting multi-scale feature maps, namely P3, P4, P5, and P6, obtained after applying SCF-FPN, are then used as input for the Region Proposal Network (RPN). The RPN generates object candidate frames based on these feature maps. Finally, we employ the cascade structure of Cascade R-CNN to filter out false detection samples at each stage, ultimately yielding the final set of detection results.

This section mainly introduces the implementation method of pest detection based on Cascade RCNN and Swin Transformer. Fig 1 illustrates the overall architecture of the network. A brief overview of the data preprocessing methods is given in Section 4.1. Then, the technical details of Feature Extraction, SCF-FPN Layer Cascaded Structure, Soft NMS, and Pseudocode are introduced in Section 4.2, Section 4.3, Section 4.4, and Section 4.5, respectively.

### 3.1 Data preprocessing

**Fig 2:**
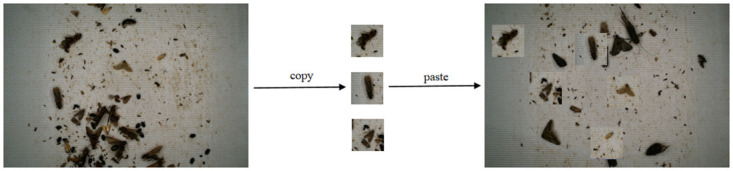
Copy and paste.

Due to the diversity of pest categories, the small amount of labeled data, and the problem of category imbalance in the labeled data, we use copy and paste to expand the dataset offline. Specifically, we cut the targets of the categories that need to be balanced, then randomly select some background images, and randomly paste these cut targets onto the background images to balance the pest categories. The effect is shown in Fig 2.

**Fig 3:**
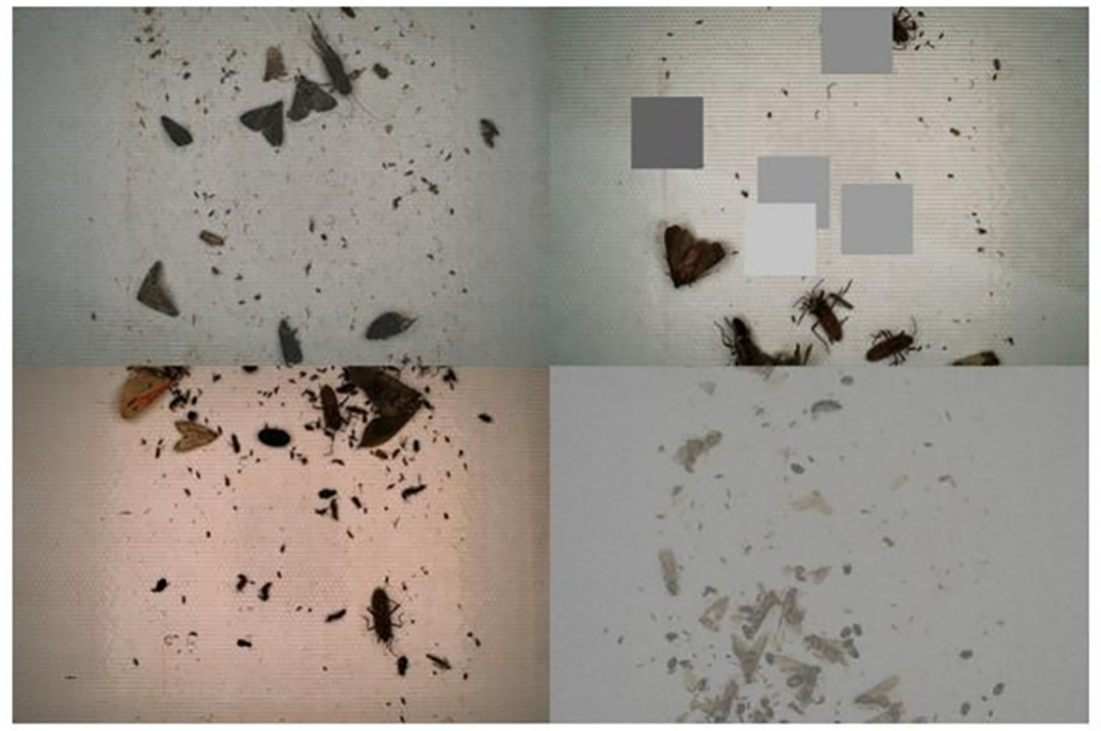
Data augmentation.

As there is uneven lighting and reflective interference between some photos, which indicates that the actual shooting environment light varies, the model needs to learn the characteristics of insects under different lighting conditions. At the same time, various pest insects have different head orientations and postures. To enhance the robustness of the target detection model and adapt it more to real-world data, we choose different strategies such as color dithering, random masking, random noise, and random enhancement of the data to achieve the effect of enhancing the diversity of the data. The effect is shown in Fig 3.

### 3.2 Feature extraction

Due to the large size of pest images, traditional CNN networks face problems such as insufficient local feature extraction, inaccurate understanding of global information, and increased computational cost. [43, 44]Therefore, we compare the experimental results of several mainstream target detection models, as shown in Table 1. It can be seen that the Cascade RCNN model with Swin Transformer as the backbone network has the highest Box-AP value. The Swin Transformer model is distinguished by its layered structure, comprising four stages. Each stage, except for the first, begins with a downsampling process of the feature maps via the Patch Merging layer. This mechanism is analogous to the layer-by-layer receptive field expansion in traditional Convolutional Neural Networks (CNNs), facilitating the acquisition of global information.

**Fig 4:**
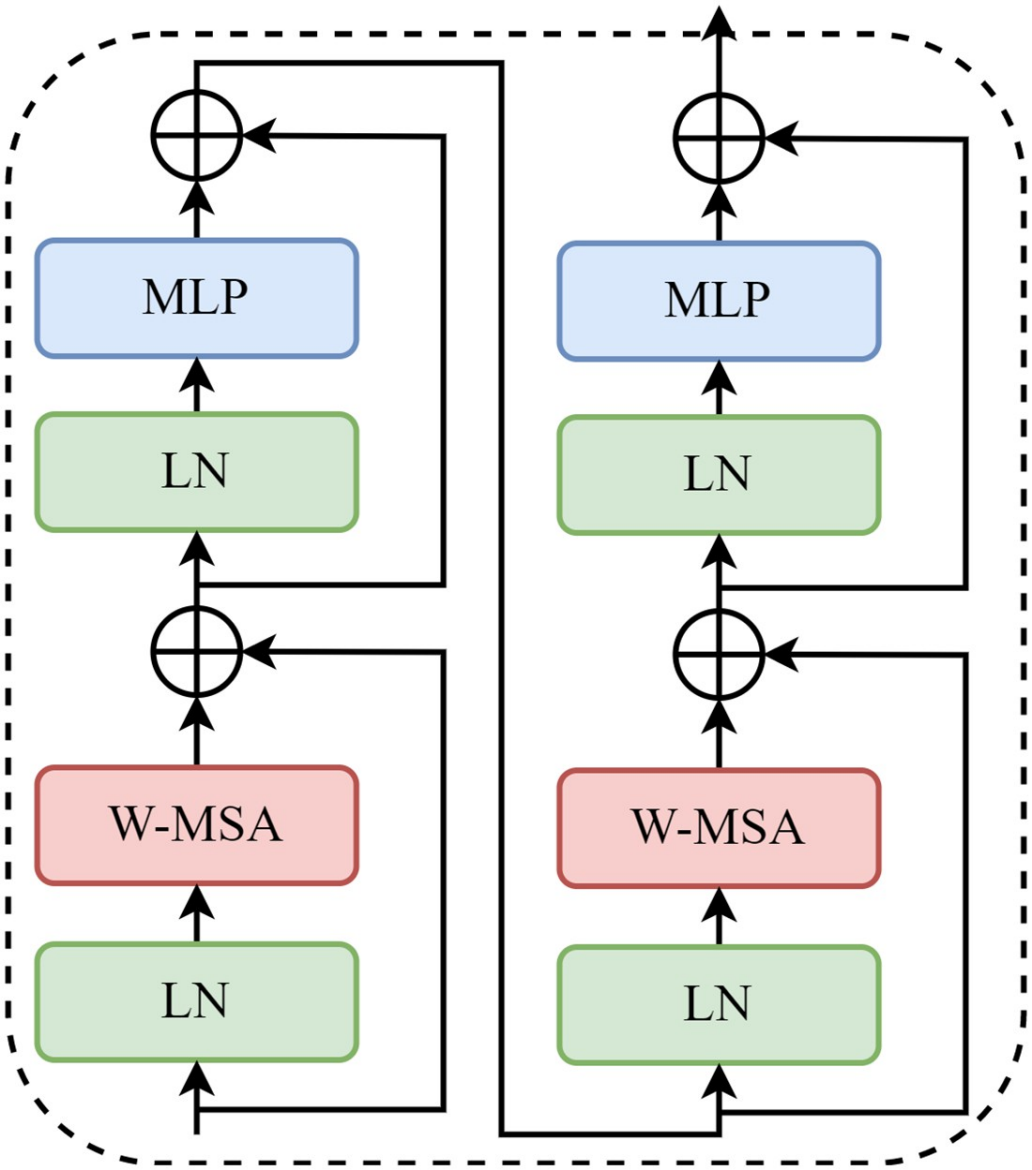
Structures of swin transformer block.

In detail, the network initially processes an input image of size H × W × 3. This image first passes through the Patch Partition layer, where a convolutional layer with a kernel size of 4 alters the output size to *H*/4 × *H*/4 × 48, effectively reducing the spatial dimensions while increasing the depth to 48 channels. Subsequently, the data enters the Linear Embedding layer, where the number of channels is adjusted to C. Each stage of the model consists of a combination of Patch Merging and several switch transformer Blocks. The Patch Merging module, positioned at the start of each stage, further downsamples the image to reduce its resolution. As illustrated in Fig 4, the Swin Transformer Block encompasses LayerNorm, Shifted Window Attention, and MLP (Multi-Layer Perceptron) modules. These components work together to process the image data effectively, ensuring local feature extraction and global information synthesis.

**Fig 5:**
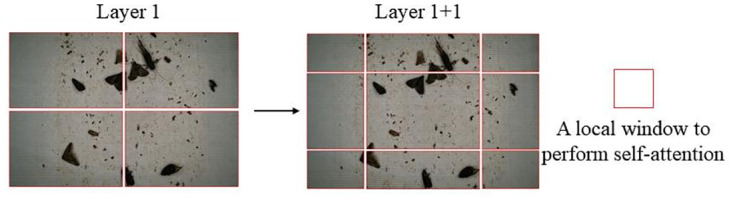
Schematic diagram of Shifted Window Attention method.

The classic Transformer architecture predominantly relies on the global computation of attention, which, while effective, results in considerably high computational complexity. The Swin Transformer introduces a novel approach to this challenge by localizing the attention computation within individual windows. This strategic confinement significantly reduces the overall computational load. Moreover, incorporating a window-shifting attention module in the Swin Transformer mitigates the risk of omission errors that traditional Convolutional Neural Networks (CNNs) might encounter when processing large-scale images. As shown in Fig 5, In the initial layer (Layer1), the model computes the self-attention mechanism within each predefined window to capture the local correlation information. In the Layer1+1 layer, the windows are shifted, changing from 4 windows to 9 windows. In the new windows, self-attention can cross the boundaries of the original windows, realizing the connections between the windows. By using the shifted window partitioning method, successive Swin Transformer blocks are computed as:

$$\begin{array}{c} {\hat{z}}^{l}=W-\text{MSA}(\text{LN}(z^{l-1}))+z^{l-1} \end{array}$$ (1)

$$\begin{array}{c} z^{l}=\text{MLP}(\text{LN}({\hat{z}}^{l}))+{\hat{z}}^{l} \end{array}$$ (2)

$$\begin{array}{c} {\hat{z}}^{l+1}=\text{SW}-\text{MSA}(\text{LN}(z^{l}))+z^{l} \end{array}$$ (3)

$$\begin{array}{c} z^{l+1}=\text{MLP}(\text{LN}({\hat{z}}^{l+1}))+{\hat{z}}^{l+1} \end{array}$$ (4)

Where ${\hat{z}}^{l}$ denotes the output of the W-MSA module and z^*l*^ denotes the output of the MLP module. W-MSA represents the window-based multi-head self-attention configured using a rule-and-shift window partition. By introducing Shifted Window Attention, Swin Transformer can capture a broader range of contextual information while maintaining a low computational complexity. This is effective for processing large-size input images or sequence data like the Pest dataset, allowing the model to understand the global context better and capture long-range dependencies. By extracting the features by Swin Transformer, we can get three feature maps: feat1, feat2, and feat1. These feature maps are the direct outputs of Swin Transformer stages 2, 3, and 4. Each feature map is associated with a different target size: feat1 corresponds to a tiny target, feat2 to a medium target, and feat3 to a large target.

### 3.3 SCF-FPN layer

**Fig 6:**
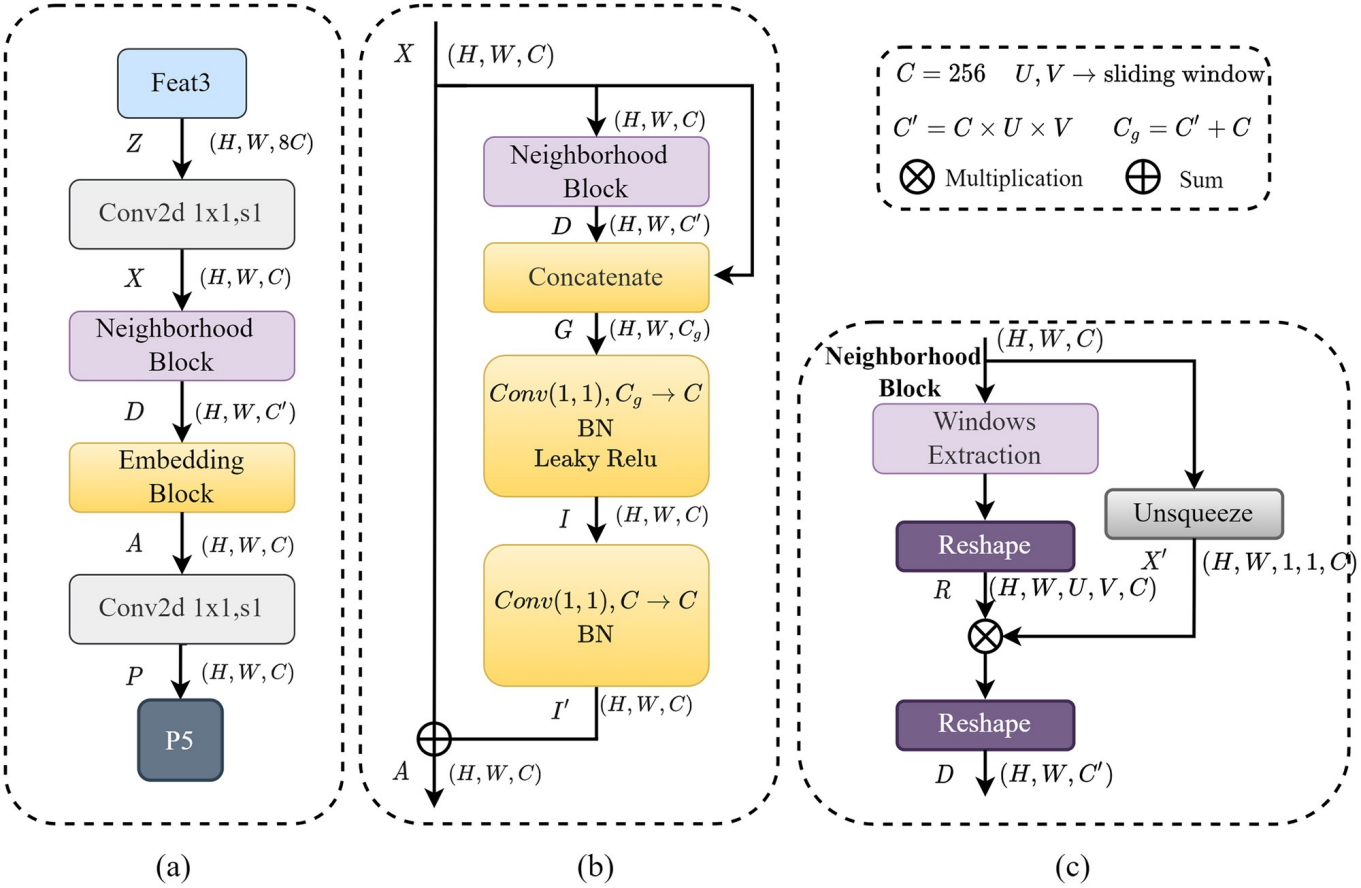
SCF-FPN layer overall architecture. (a) Representation of the process from feat3 to P5 (b) Representation of the detailed structure of SCF (c) Representation of the detailed structure of Neighborhood Block.

Since many pests have different body sizes, we designed the SCF-FPN layer to identify pest characteristics at different scales, as shown in Fig 6. Specifically, the SCF self-attention network includes two main learnable modules: Neighborhood Block and Embedding Block.

#### 3.3.1 Neighborhood Block

In the previous section, we used Swin Transformer to obtain the essential features Z(feat1,feat2,feat3); to get a more compact representation, we used the downsampling convolution module to reduce the channel dimension of each feature map to 256, and the resulting downsampled features are collectively referred to as X. To further extract the localized features, we use the sliding fixed-window operation W [45], thus obtaining the regional features *R*.

$$\begin{array}{c} R=\text{reshape}\left(W(X)\right)\in R^{H\times W\times U\times V\times C} \end{array}$$ (5)

where *U* and *V* are the height and width of the window. In our experiments, we chose symmetric windows, i.e., (*U*, *V*) ∈ {(3, 3), (5, 5), (7, 7)} (Here we use (*U*, *V*) ∈ (7, 7), (*U*, *V*) ∈ (5, 5) and (*U*, *V*) ∈ (3, 3) for the size of the sliding window for feat1, feat2 and feat3 respectively). We use the Hadamard product to compute the autocorrelation tensor D to efficiently capture the internal correlation between different dimensions in the original image and enhance the extracted features.

$$\begin{array}{c} D=\text{reshape}\left(R\times X^{\prime }\right)\in R^{H\times W\times C^{\prime }} \end{array}$$ (6)

where *X*′ represents the downsampled feature *X* that has undergone dimensionality augmentation and *C*′ = *U* × *V* × *C*. The autocorrelation tensor D treats the neighborhood window as part of the channel features and can more efficiently capture local relations and self-similarity. The specific details of the Neighborhood Block are shown in Fig 6(c).

#### 3.3.2 Embedding Block

While neighborhood blocks can learn correlations between different features, they lack the local semantic cues the original convolutional features provide. We use a simple fusion. step to create a contextual feature-aware semantic representation and an Embedding Block to capture various attributes of semantic objects. We specialize in stitching X and D together to create contextual semantic features $G\in R^{H\times W\times C_{g}}$ as shown below.

$$\begin{array}{c} G_{\left(i,j\right)}={\left[X_{\left(i,j\right)}^{T},D_{\left(i,j\right)}^{T}\right]}^{T} \end{array}$$ (7)

In this equation, *G*_(*i*, *j*)_ refers to the contextual semantic features at a specific location (*i*, *j*) in the image, *C*^*g*^ = *C*′+ *C*. After the Concatenate operation, we use two convolutional layers to remap the merged feature map to a higher-level feature space. Specifically, the first convolutional layer is used to compress and learn a new representation of the integrated features. In contrast, the second convolutional layer is used to refine these features further and recover the feature count. Finally, we residualized its output to obtain the final enhanced feature *A* (the specific flow of the Embedding Block is shown in Fig 6(b).

$$\begin{array}{c} \text{I}=F_{1\times 1}(G)\in R^{H\times W\times C} \end{array}$$ (8)

$$\begin{array}{c} {\text{I}}^{\prime }=F_{1\times 1}(I)\in R^{H\times W\times C} \end{array}$$ (9)

$$\begin{array}{c} A=X+I^{\prime } \end{array}$$ (10)

where $F_{1\times 1}$ denotes a 1 × 1 convolutional layer. The output attention feature A is passed through a 3 × 3 convolutional layer to get the final output features P3, P4, P5, and P6, which usually correspond to feature maps of different resolutions and can be used to detect objects of various sizes.

### 3.4 Cascaded structure and soft NMS

We input the multi-scale feature maps (P3, P4, P5, P6) obtained after SCF-FPN into RPN and generate them into object candidate frames. Pest detection for small targets is often challenging due to different pests’ unique appearance and characteristics. We utilize the cascade structure of Cascade R-CNN to filter out false detection samples at each stage so that the model can focus on smaller targets, thus improving the detection of small targets. Each stage in the Cascade R-CNN framework applies increasingly stringent IoU thresholds to refine and classify the pre-selected boxes. Specifically, we set these IoU thresholds at 0.5, 0.6, and 0.7 for each successive stage. The final classification result is obtained by summing and averaging the outputs of C1, C2, and C3. The result of the detected frame is the detected frame output by B3 as the final detection result.

In addition, to eliminate redundant, overlapping detection frames, we added the Soft NMS (Soft NMS) strategy. Unlike traditional NMS, Soft NMS will not directly delete detection frames with high overlap based on the degree of overlap with the highest-scoring detection frame. Reduce its score. Soft NMS will first find the detection frame with the highest score and then calculate the overlap (IOU) of all other detection frames with this detection frame. Soft NMS will Overlap for each detection frame whose overlap exceeds a certain threshold to reduce its score. In this way, even if the score of a specific detection frame is reduced, as long as its score is still higher than a certain threshold, it can still be retained. Its main improvements are as follows:

$$\begin{array}{c} s_{i}=\{\begin{array}{cc} s_{i}, & \text{iou}(M,b_{i})<N_{t}\\ s_{i}(1-\text{iou}(M,b_{i})), & \text{iou}(M,b_{i})\geq N_{t} \end{array} \end{array}$$ (11)

In the equation, *s*_*i*_ represents the confidence score of the I detection box, $\text{iou}(M,b_{i})$ represents the intersection over union (IoU) between the current main box M and I detect on box *b*_*i*_, and *N*_*t*_ represents the threshold for the overlap ratio used to determine whether to adjust the score. This way, Soft NMS can better preserve neighboring high-quality detection results, thereby improving the performance of pest detection.

**Algorithm 1:** Cascade RCNN Combined with Swin Transformer Pseudocode

**Input**: Input image, Number of stages in cascade (3), IoU thresholds for each stage

**Output:** Final object detection boxes with class labels and scores

// Extract feature maps for large, medium, and small objects using Swin Transformer

*feat*1, *feat*2, *feat*3 ← Swin Transformer(Input image)

// Input the feature maps into SCF-FPN to get multi-scale features *P*3, *P*4, *P*5, *P*6 ← SCF-FPN(*feat*1, *feat*2, *feat*3)

// Initialize proposals from Region Proposal Network (RPN) *proposals* ← RPN(*P*3, *P*4, *P*5, *P*6)

**for** *i* ← 1 **do**

 // Refine detection boxes at each stage of the cascade

 // Adapt the detector to the current IoU threshold

 *adjusted*_*detector* ← AdjustDetector(detector, IoU_thresholds[*i*])

 // Apply the adjusted detector to the proposals

 *refined*_*proposals*, *scores*, *labels* ← Detect(*adjusted*_*detector*, *proposals*, *P*3, *P*4, *P*5, *P*6)

 // Update proposals for the next stage

 *proposals* ← *refined*_*proposals*

 **if** *i* = 3 **then**

  // Final stage, output the detection results

  **return** *refined*_*proposals*, scores, labels

### 3.5 Pseudocode

We have added a detailed pseudo-code to let everyone understand our Cascade RCNN Combined with the Swin Transformer model more clearly. Algorithm 1 shows the pseudo-code of the entire model: Input image, Number of stages in cascade (3), and IoU thresholds for each stage to output the Final object detection boxes with class labels and scores.

## 4 Experiment and analysis

In this section, the pest dataset used is described in detail, and the performance of our method on the pest dataset is evaluated and compared with other methods. In addition, ablation experiments will be performed to verify the effectiveness of the main modules.

### 4.1 Dataset

The pest data set in this article uses the 10th “Teddy Cup” competition data set. The original data contains 3015 pictures, with 28 different categories of pests. According to the information provided in the picture insect location details table, there are 1027 of them. Images containing targets, 1186 background images without pests, and 802 images used to test the final results. Due to the small amount of available data, we expanded it using the data preprocessing method in Section 4.1. The original data target of 1027 was raised to 4515 targets. The number of images in the initial training set is 2213, and the expanded training set is 5701. Details are given in Table 2. The details of the expanded data set are shown in Table 3.

**Table 2:** Pest dataset details.

Method	Target image	Background image	Total train	Total test	Sum
Original	1027	1186	2213	802	3015
Expansion	4515	1186	5701	802	6503

**Table 3:** Original and expanded datasets explained.

Id	Name	Original number	Expansion number
6	Sesamia inferens	20	164
7	Chilo suppressalis	90	165
8	Rice case worm	18	165
9	White backed planthopper	120	165
10	Nilaparvata	150	163
25	Cutworm	5	162
41	Gryllotalpa spps	15	165
105	Armyworm	27	163
110	Beet webworm	6	167
115	Spodoptera exigua	17	161
148	Sirthenea flavipes	59	166
156	Grey mulberry hairy caterpillar	242	242
222	Cotton boll worm	20	163
228	Athetis lepigone	21	165
235	Cabbage moth	5	162
256	Cricket	87	165
280	Euproctis chrysorrhoea	40	163
310	Rice green semilooper	6	161
387	Calothysanis comptaria Walker	3	136
392	Pyralid	6	162
394	Athetis lineosa	5	162
398	Spoladea recurvalis	15	163
401	Endotricha	7	164
402	Diaphania indica	5	135
430	Maruca testulalis Geyer	3	120
480	Caddisfly	18	165
485	Holotrichia oblita	12	146
673	Staurophora celsia	5	135
sum	28	1027	4515

### 4.2 Experimental details

This paper conducts experiments using Pytorch 1.9 deep learning framework in an environment based on NVIDIA 3090 GPU and Intel i7 9700K CPU. The input image size is 1280*1280 in the experiment with channel C = 256. Sliding window size in SCF-FPN use (*U*, *V*) ∈ (7, 7), (*U*, *V*) ∈ (5, 5) and (*U*, *V*) ∈ (3, 3) for the size of the sliding window for feat1, feat2 and feat3 respectively. In terms of the optimizer, this paper uses SGD optimizer with momentum set to 0.9, learning rate starting from 0.01, and decay factor of 0.05. The training on the pest dataset is performed for 100 epochs, with the number of samples in each training batch being 64. On the 80th and 90th epoch, the learning rate was reduced by 0.1 using a learning rate decrease strategy.

### 4.3 Results

**Fig 7:**
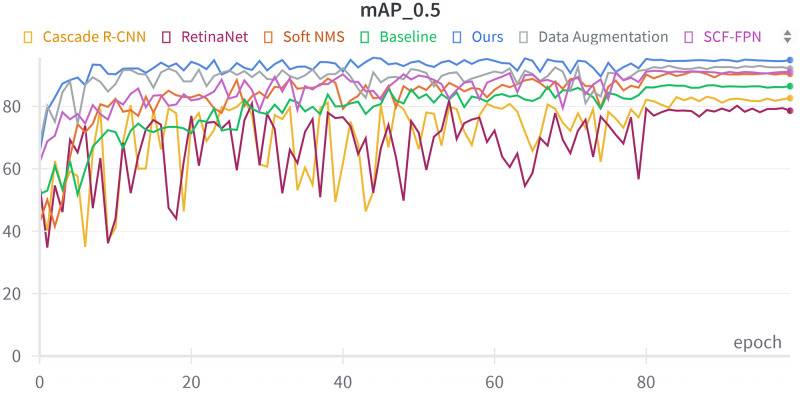
Effect of adding different modules on mAP_0.5.

**Fig 8:**
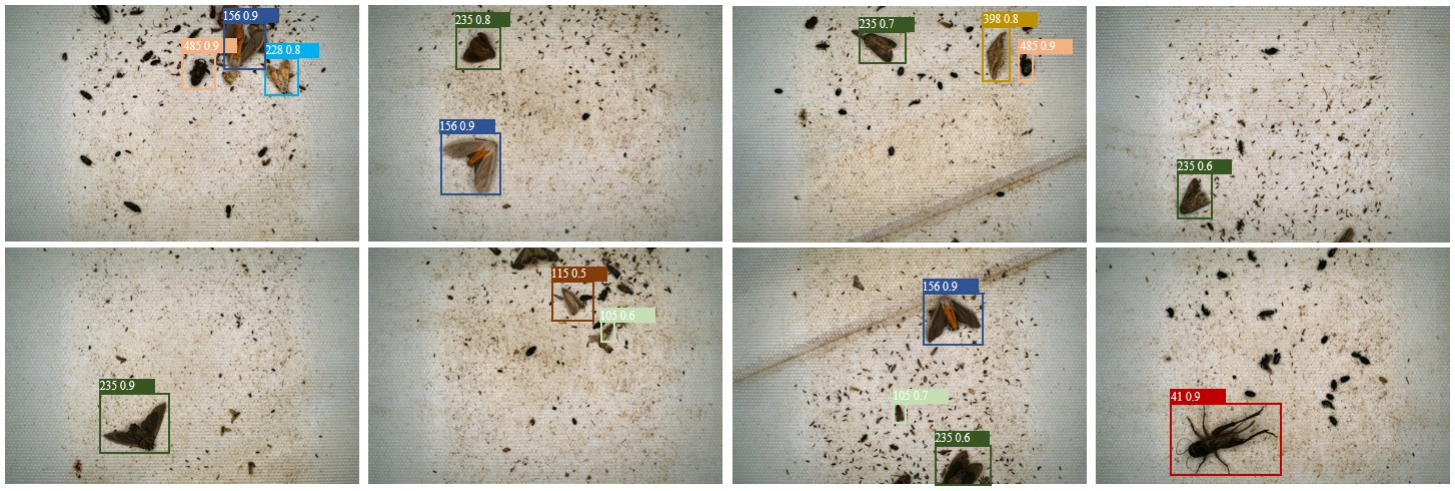
Prediction results for some of the pests in our model.

According to the experimental results (see Table 4), the Cascade RCNN model using Swin Transformer as the backbone network achieves sub-optimal performances of 80.4%, 86.4%, and 86.1% in terms of accuracy, recall, and average precision without other changes. Our method performs best by expanding the dataset and introducing the improved SCF-FPN layer and Soft NMS. Compared to the Cascade RCNN model using X-101-64x4d-FPN as the backbone network, the improved model shows an improvement of 15.4%, 11.3%, and 12.5% in accuracy, recall, and average precision, respectively. It can also be seen from Fig 7 that our mAP_0.5 curves are significantly smoother and converge faster than the other comparative methods, achieving a steady and consistent improvement over a small number of training rounds. In contrast, the mAP_0.5 curves of RetinaNet and the Cascade R-CNN based on X-101-64x4d-FPN exhibit significant fluctuations and slower convergence. In addition, these models reached low peaks in recognition correctness, implying limitations in their ability to detect and classify pests accurately, especially in challenging scenarios. Fig 8 illustrates the outstanding detection results achieved by the enhanced model on the pest dataset. The model depicted in the figure demonstrates the capability to identify a wide range of pests. Notably, it exhibits accurate identification not only for pests with a substantial number of samples but also for those with fewer samples, such as instances 41, 115, and 235. This superiority can be attributed to several factors. Firstly, after data augmentation, Swin Transformer serves as the backbone network, enabling better extraction of key features. Additionally, the introduction of the SCF-FPN layer enhances the model’s focus on pests of varying sizes. Lastly, the application of Soft NMS effectively improves the performance of pest detection by mitigating overlapping detection frames.

**Table 4:** Comparison with other target detection methods on pest datasets.

Method	Backbone	Precision	Recall	mAP_0.5
Mask R-CNN	X-101-64x4d-FPN	75.7	78.4	79.2
Faster	X-101-64x4d-FPN	75.2	77.6	78.7
RetinaNet	X-101-64x4d-FPN	74.5	77.2	77.8
Cascade R-CNN	X-101-64x4d-FPN	77.1	80.5	81.2
Cascade R-CNN	Swin-S-FPN	80.4	86.4	86.1
**Ours**	Swin-SCF-FPN	**92.5**	**91.8**	**93.7**

### 4.4 Ablation studies

To verify the effect of the main modules, we use Swin-S-FPN without any added modules as the baseline model and introduce data expansion, SCF-FPN, and Soft NMS one by one to test the effect of different modules on the performance. According to the results in Table 5, the data expansion module significantly improves the original baseline model when a module is introduced alone. The accuracy, recall, and average precision increased by 7.4%, 3.2%, and 5.1%, respectively. SCF-FPN also improves performance when used alone, albeit to a lesser extent than data scaling, emphasizing its role in better feature representation and scale consistency.

**Table 5:** Ablation experiments with data expansion, SCF-FPN, and soft NMS.

Data Expansion	SCF-FPN	Soft NMS	Precision	Recall	mAP_0.5
×	×	×	80.4	86.4	86.1
✓	×	×	87.8	89.6	91.2
×	✓	×	85.2	88.6	89.2
×	×	✓	84.7	87.4	88.7
✓	✓	×	91.2	90.8	92.6
×	✓	✓	88.1	89.3	91.4
✓	×	✓	89.4	90.4	92.2
✓	✓	✓	**92.5**	**91.8**	**93.7**

Further, when data expansion and SCF-FPN are introduced, the model reaches sub-optimal accuracy, recall, and average precision stages. This indicates that combining data expansion and SCF-FPN plays a role in multi-scale feature fusion, improving small target detection and solving the object scale variation problem. The network performance is optimal when all three modules are introduced. The accuracy, recall, and average precision are improved by 12.1%, 5.4%, and 7.6%, respectively, further validating the feasibility of the improved algorithm. Fig 7 demonstrates how adding different modules improves the baseline model regarding mAP _ 0.5.

To thoroughly assess the influence of sliding window sizes on the performance of the SCF-FPN network, we conducted a series of experiments with different window configurations. Three control groups, A, B, and C, were established, representing the Neighborhood Block in which sliding windows of sizes (3, 3), (5, 5), and (7, 7) were respectively used. Experimental group D, on the other hand, employed the original setup: using window size (7,7) for feat1, (5,5) for feat2, and (3,3) for feat3.

**Fig 9:**
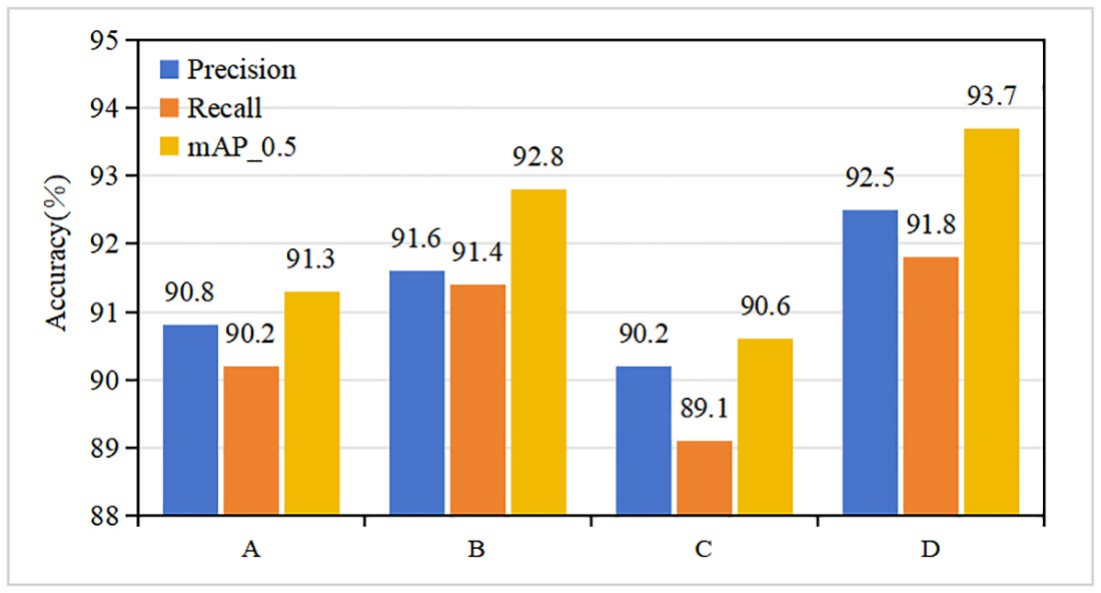
Impact of different window takes on network performance in Neighborhood Block.

As depicted in Fig 9, the performance metrics exhibit notable variations across these window sizes. Group D demonstrates the best overall network performance with the mixed window size configuration. This superior performance indicates the model’s ability to capture and process varying spatial contexts depending on the feature map scale. Group B, employing a consistent (5, 5) window size, follows closely in terms of performance, suggesting that a moderate window size can strike a balance between capturing local details and maintaining sufficient contextual awareness for most features.

However, groups A and C, with the most petite (3, 3) and most significant (7, 7) window sizes, respectively, show varying degrees of performance degradation. This outcome implies that the model’s sensitivity to the spatial context captured by the sliding window highly depends on the window size. Notably, the smaller window in Group A may limit the model’s ability to capture broader contextual information. In contrast, the larger window in Group C could overlook finer details crucial for accurate feature recognition.

## 5 Conclusions

Our research presents a robust solution to the critical challenge of agricultural pest detection, a vital issue in farming that leads to significant economic and food safety concerns. We developed a novel two-stage target detection framework by integrating Cascade RCNN with the Swin Transformer model, specifically designed to address the diversity of pests and the limited availability of labeled images. First, we extend the dataset using random cut-and-paste and traditional online enhancement techniques to enhance the learning capability of the model. Then, we performed basic feature extraction by swim-transform and used the SCF-FPN layer, which was improved. Finally, we introduced soft NMS for further optimization. Our algorithm achieves significant results regarding accuracy, recall, and mean accuracy precision (mAP_0.5), respectively, for a detection task containing 28 pest species. The results show that applying deep learning methods to the detection and identification of pests and diseases in farmland can effectively improve the accuracy of pest identification, avoid the abuse and misuse of pesticides, reduce pesticide residues on agricultural products, and improve the ecological environment of farmland.

However, even if the data set is expanded through random cut-and-paste and online enhancement techniques, there may still be a class imbalance or sparse samples, especially for some rarer pest species. This may affect the model’s generalization ability and performance. Current models require significant computing resources and time to complete detection tasks, which may be unacceptable for real-time monitoring. Therefore, the next step of research work will focus on improving the quality and quantity of the data set and optimizing the real-time performance of the model.

## Supporting information

S1 Fig Pictures of some farmland pests. (ZIP)
