# Supplementary figures and images for: Farmland pest recognition based on Cascade RCNN Combined with Swin-Transformer
Source: PLoS One. 2024 Jun 6;19(6):e0304284. doi: 10.1371/journal.pone.0304284 (PMC11156394; doi:10.1371/journal.pone.0304284)

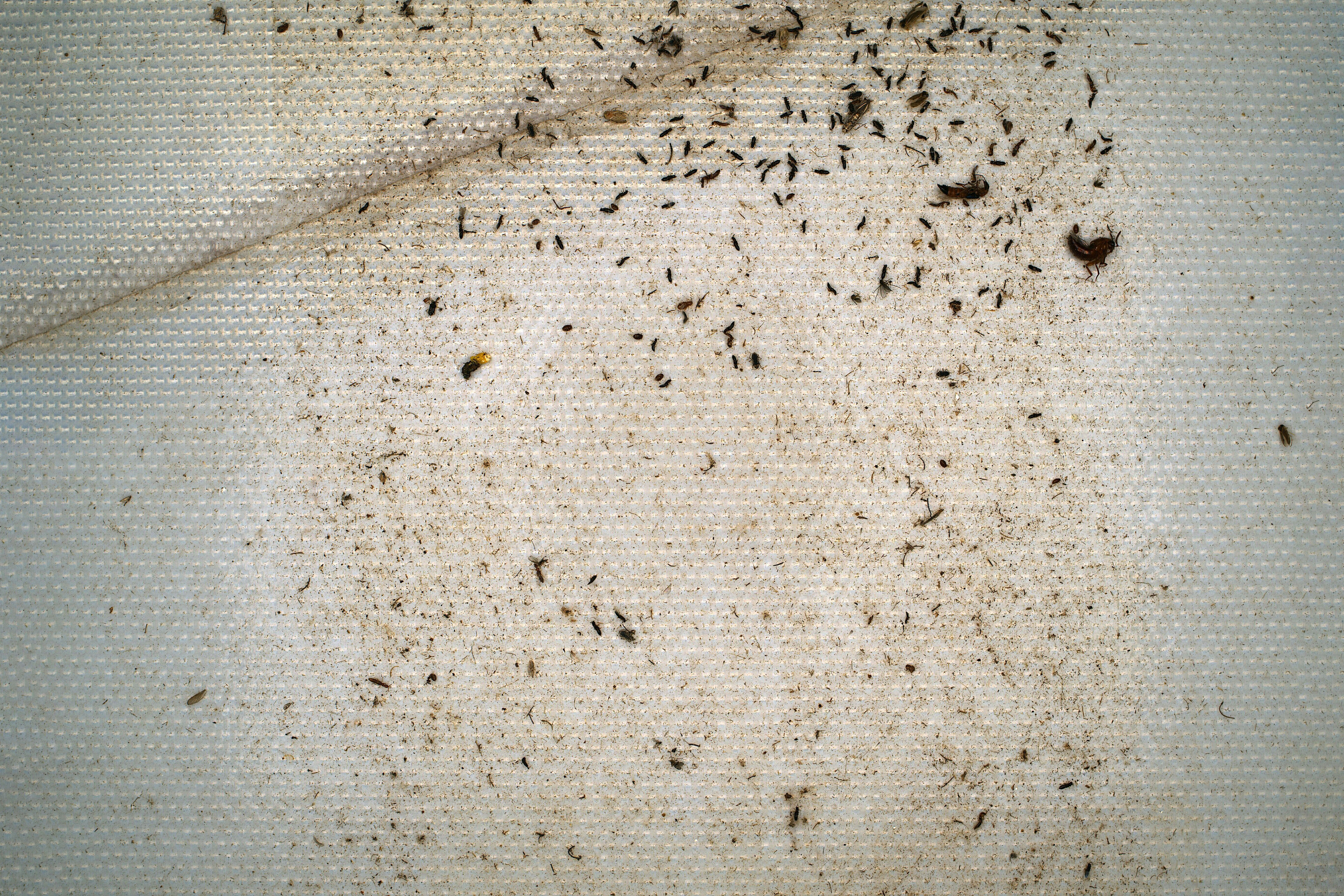

Supplement: S1 Fig — (ZIP) [file pone.0304284.s001.zip › 0001.jpg]

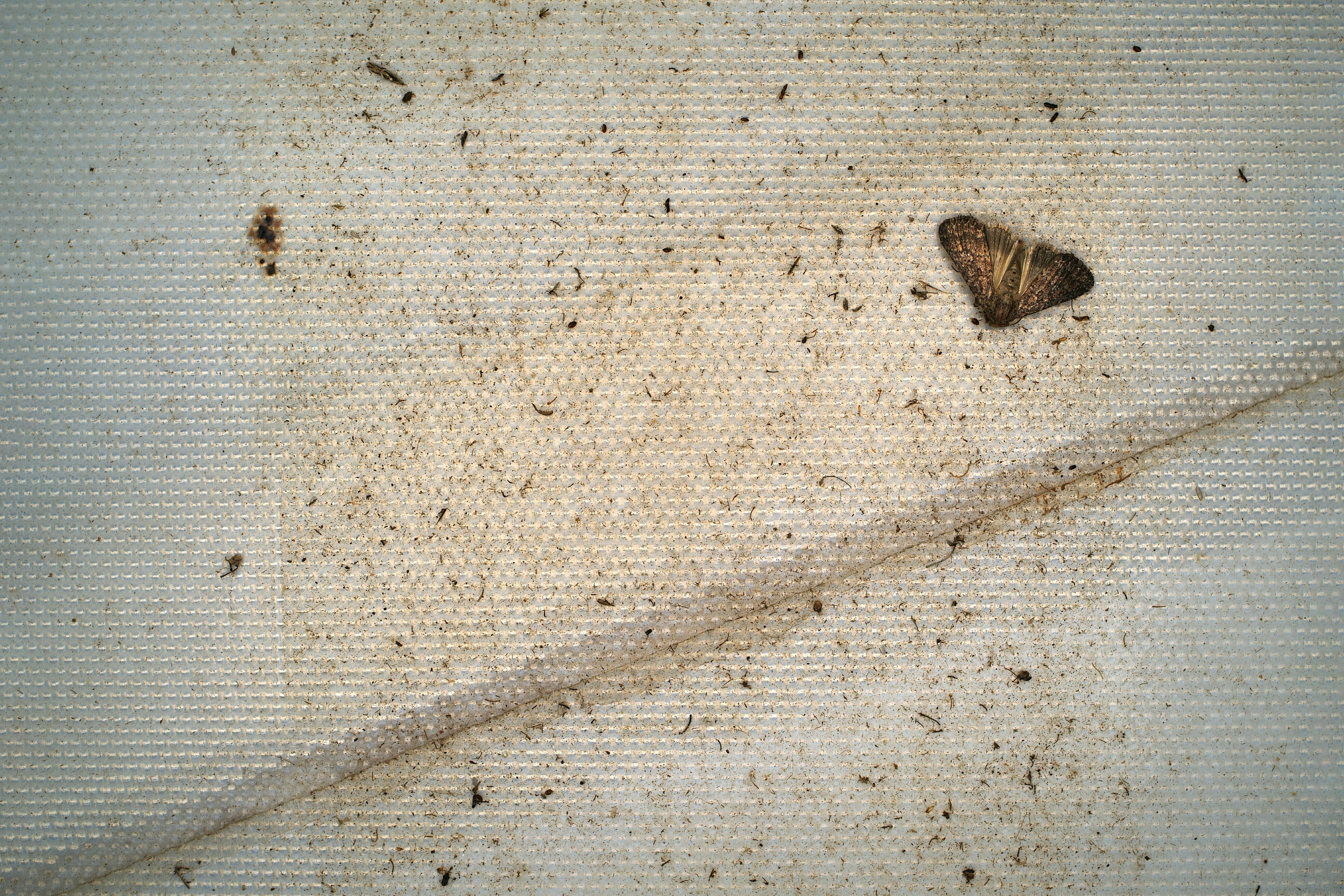

Supplement: S1 Fig — (ZIP) [file pone.0304284.s001.zip › 0002.jpg]

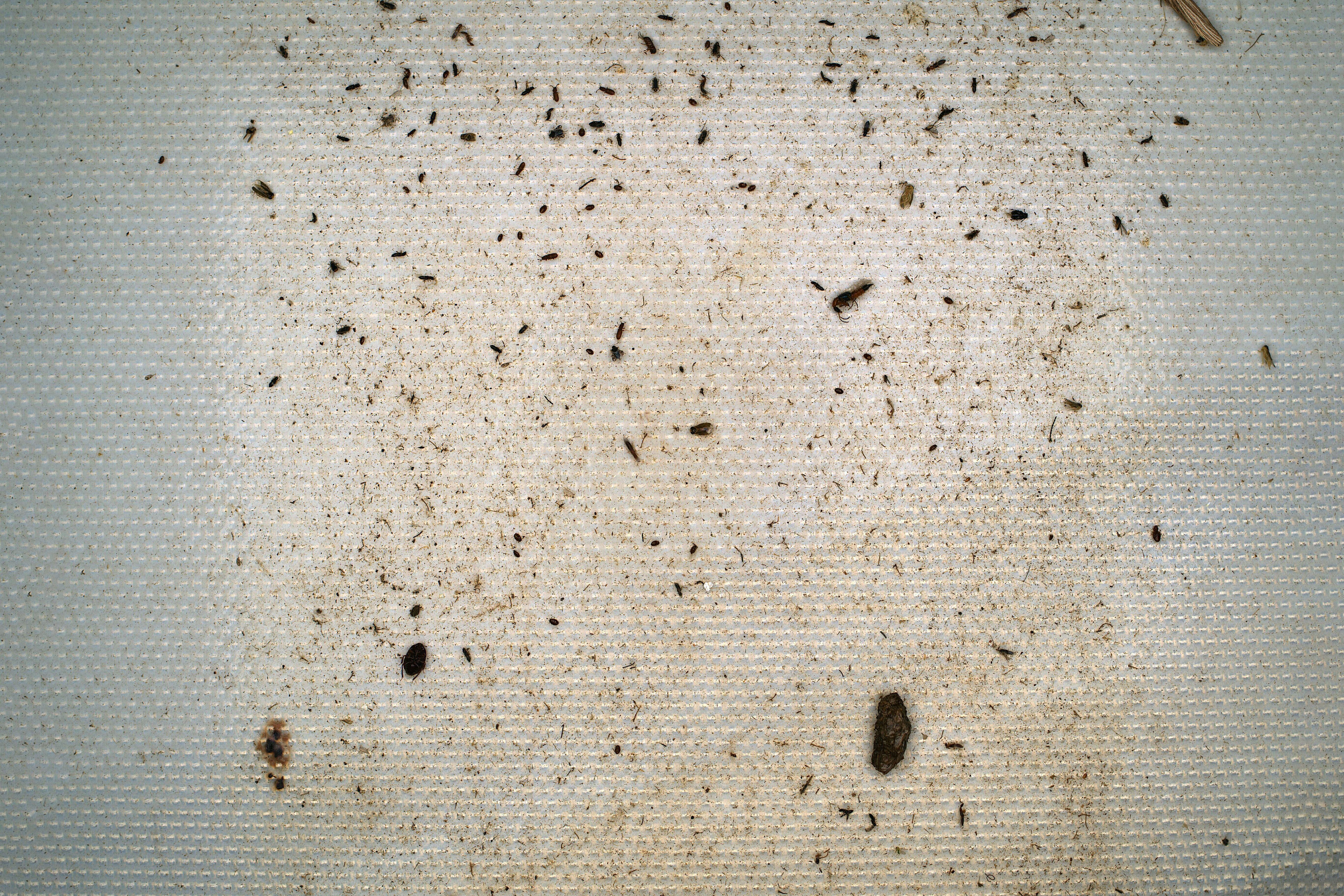

Supplement: S1 Fig — (ZIP) [file pone.0304284.s001.zip › 0003.jpg]

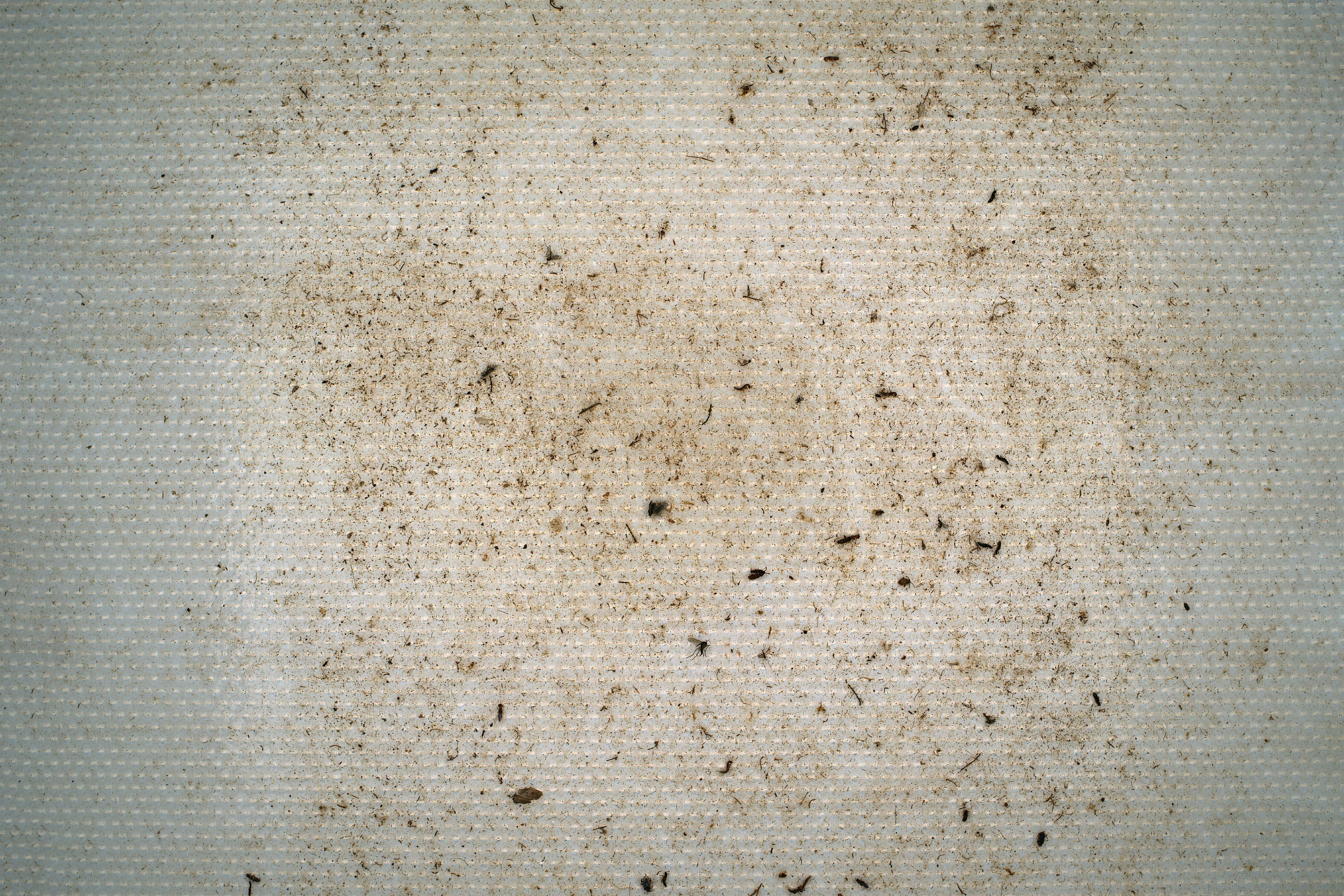

Supplement: S1 Fig — (ZIP) [file pone.0304284.s001.zip › 0004.jpg]

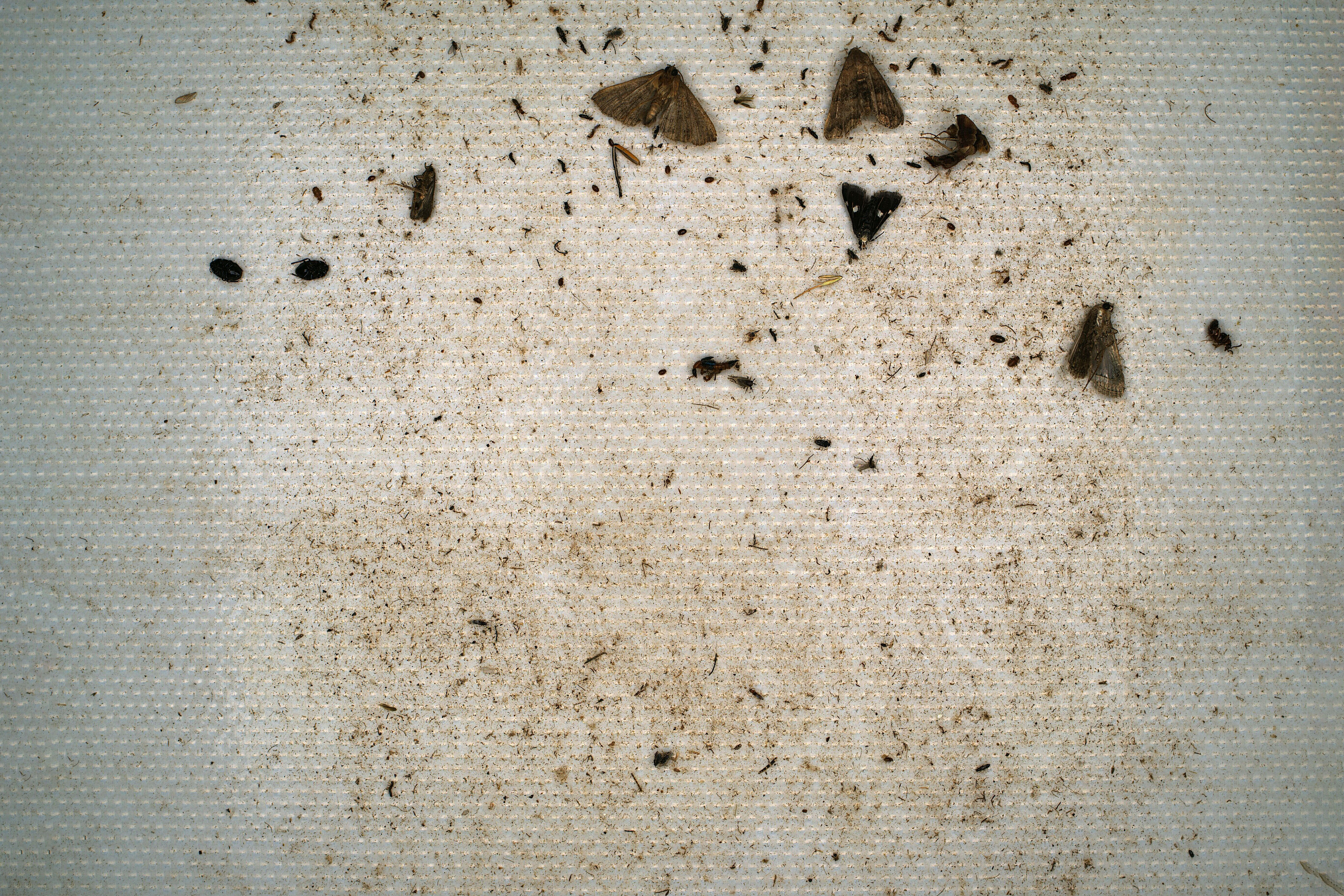

Supplement: S1 Fig — (ZIP) [file pone.0304284.s001.zip › 0005.jpg]

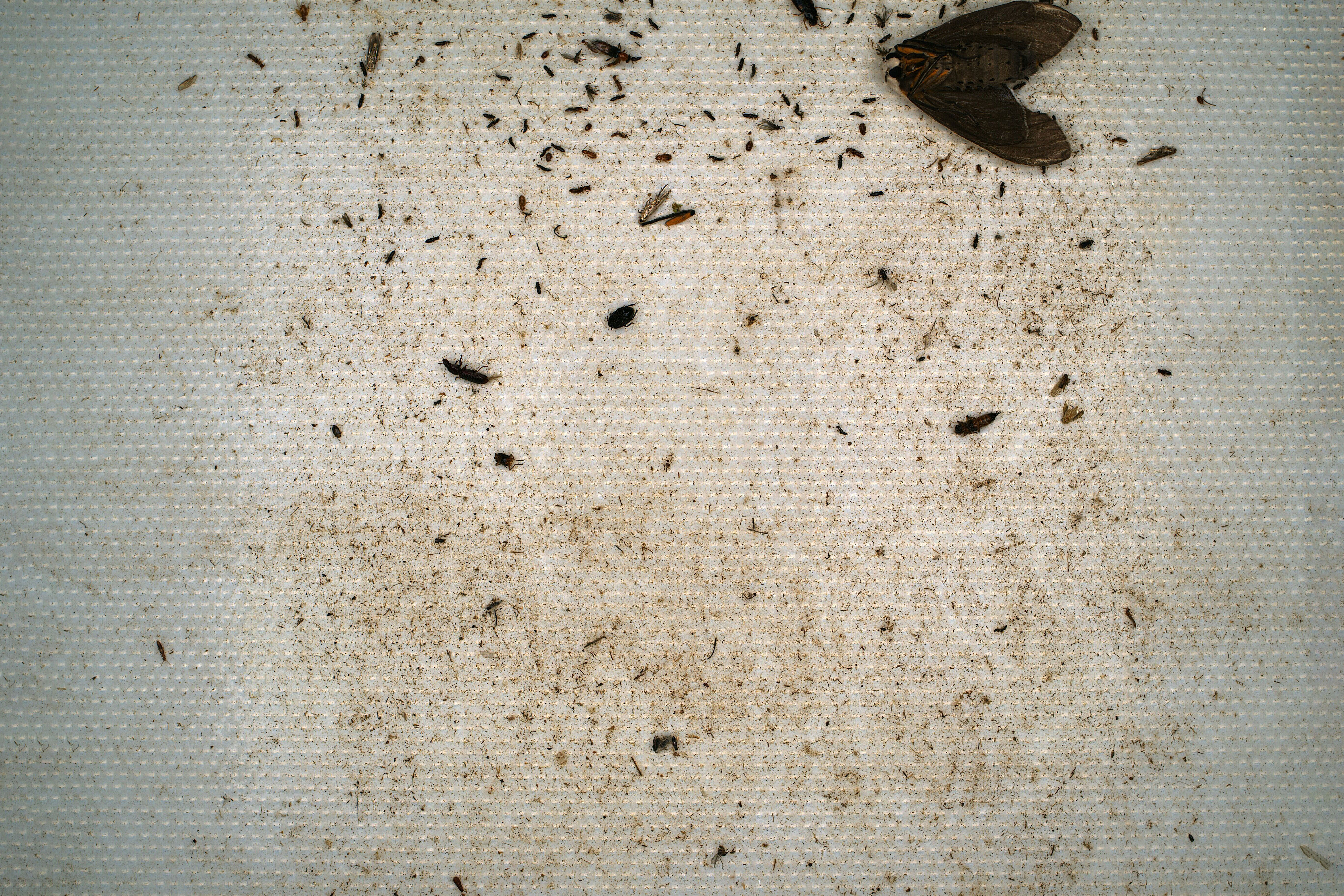

Supplement: S1 Fig — (ZIP) [file pone.0304284.s001.zip › 0006.jpg]

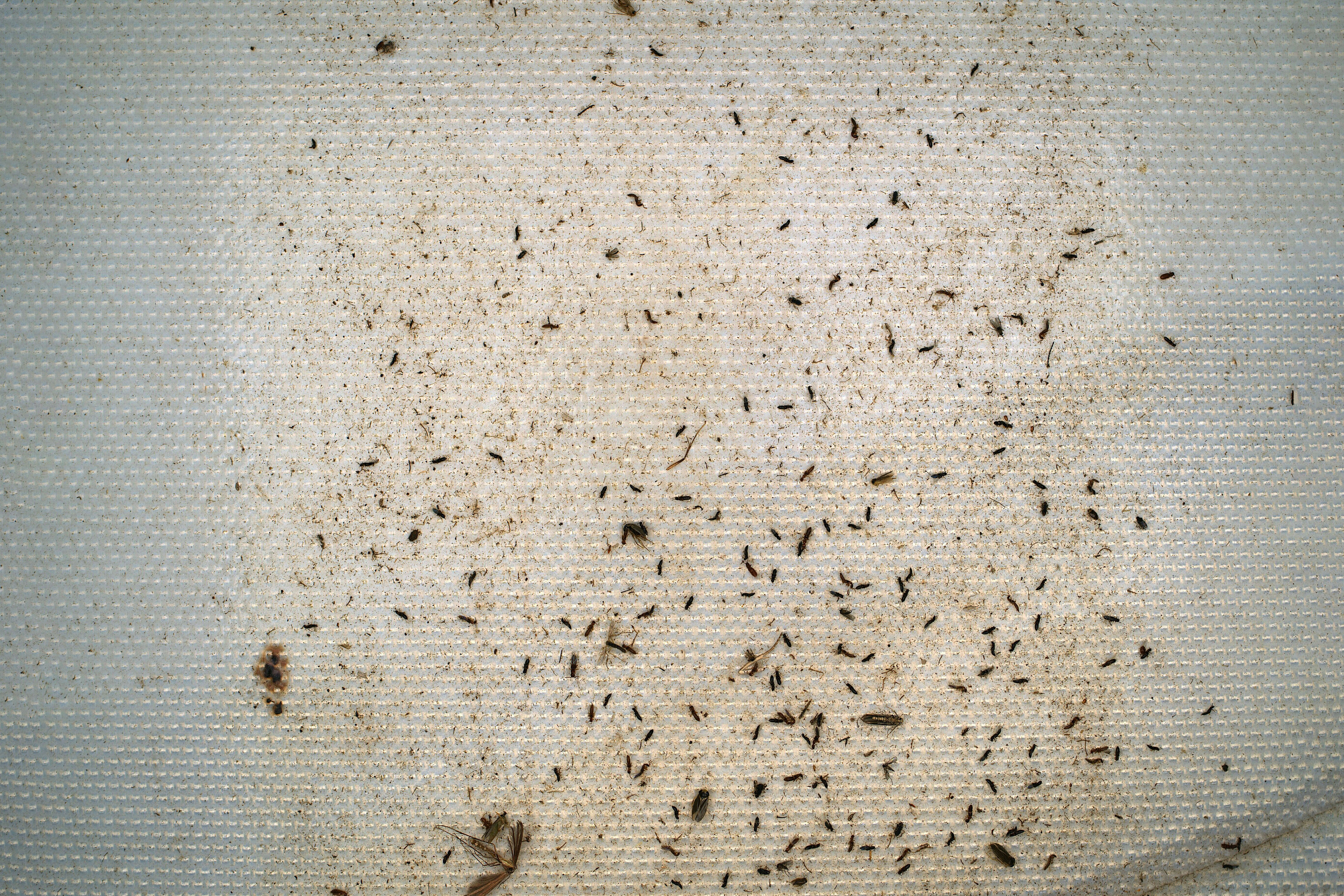

Supplement: S1 Fig — (ZIP) [file pone.0304284.s001.zip › 0007.jpg]

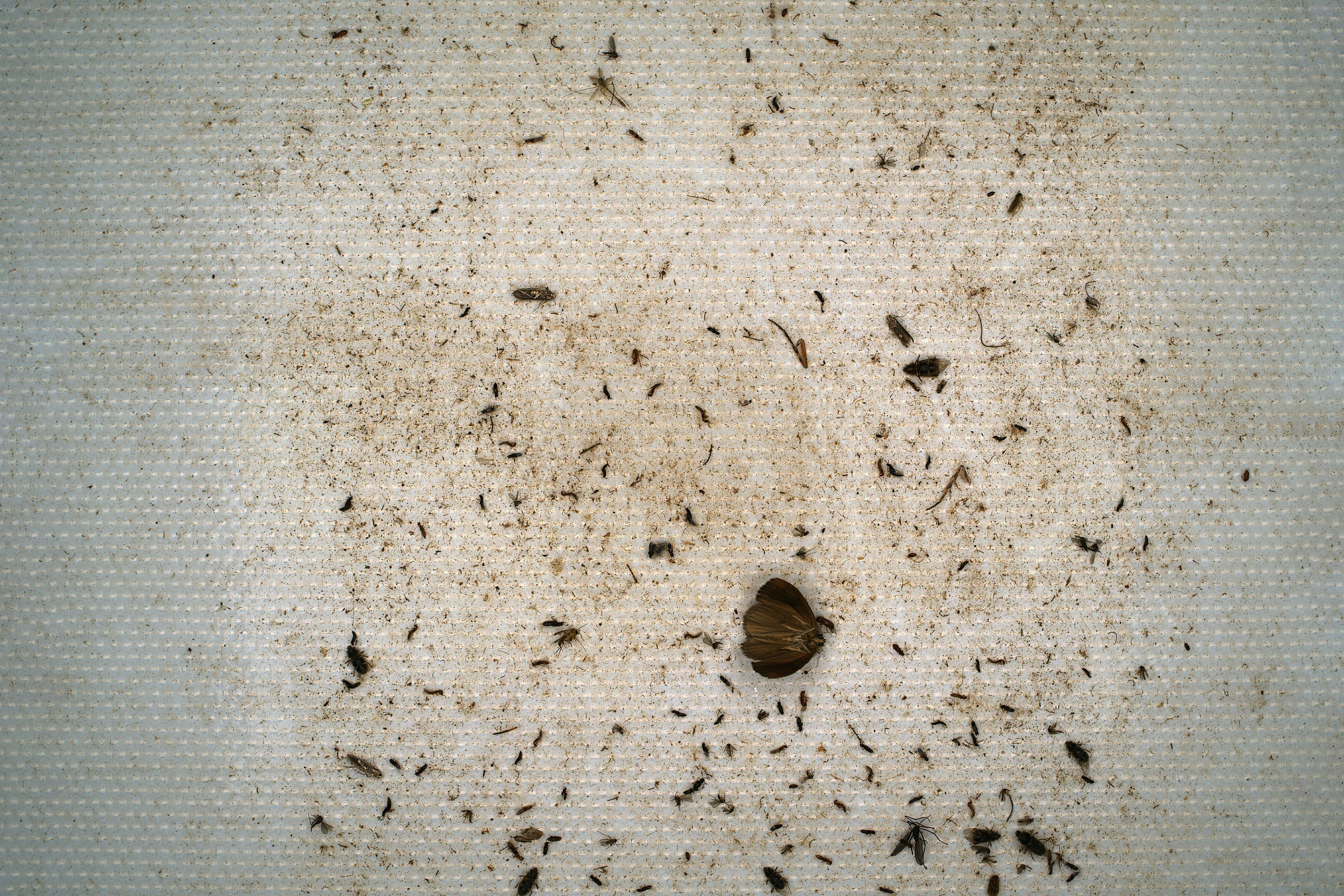

Supplement: S1 Fig — (ZIP) [file pone.0304284.s001.zip › 0010.jpg]

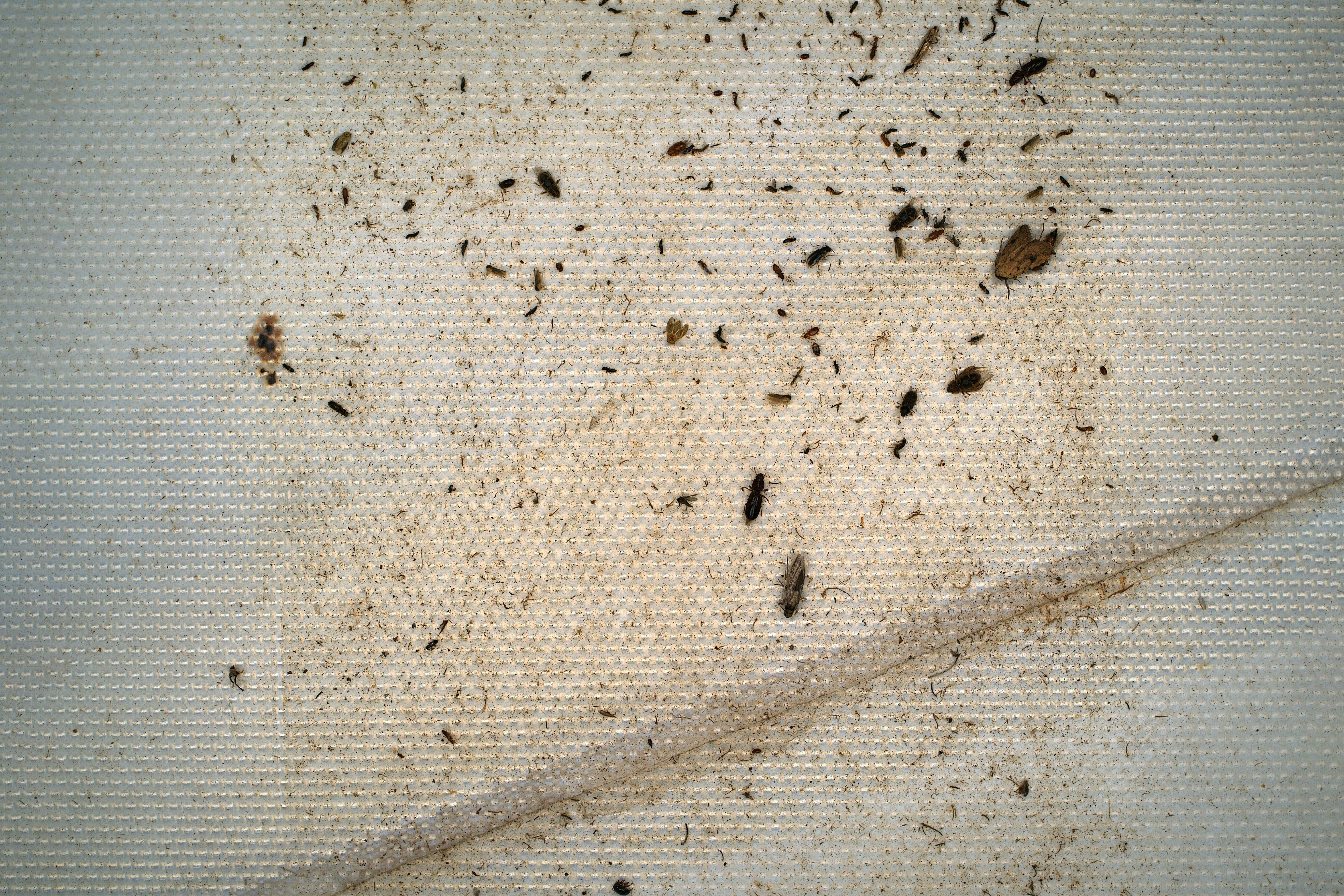

Supplement: S1 Fig — (ZIP) [file pone.0304284.s001.zip › 0011.jpg]

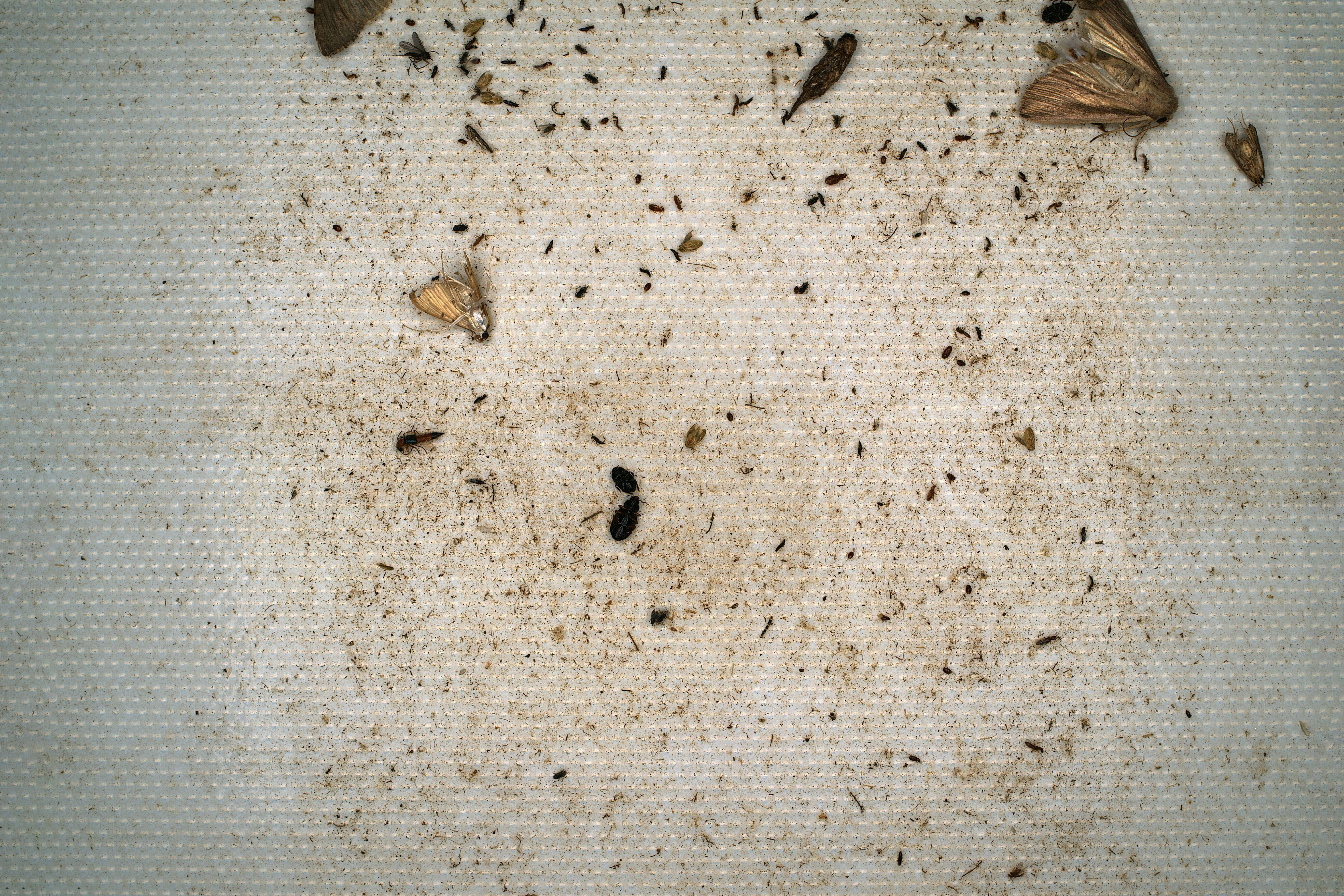

Supplement: S1 Fig — (ZIP) [file pone.0304284.s001.zip › 0012.jpg]

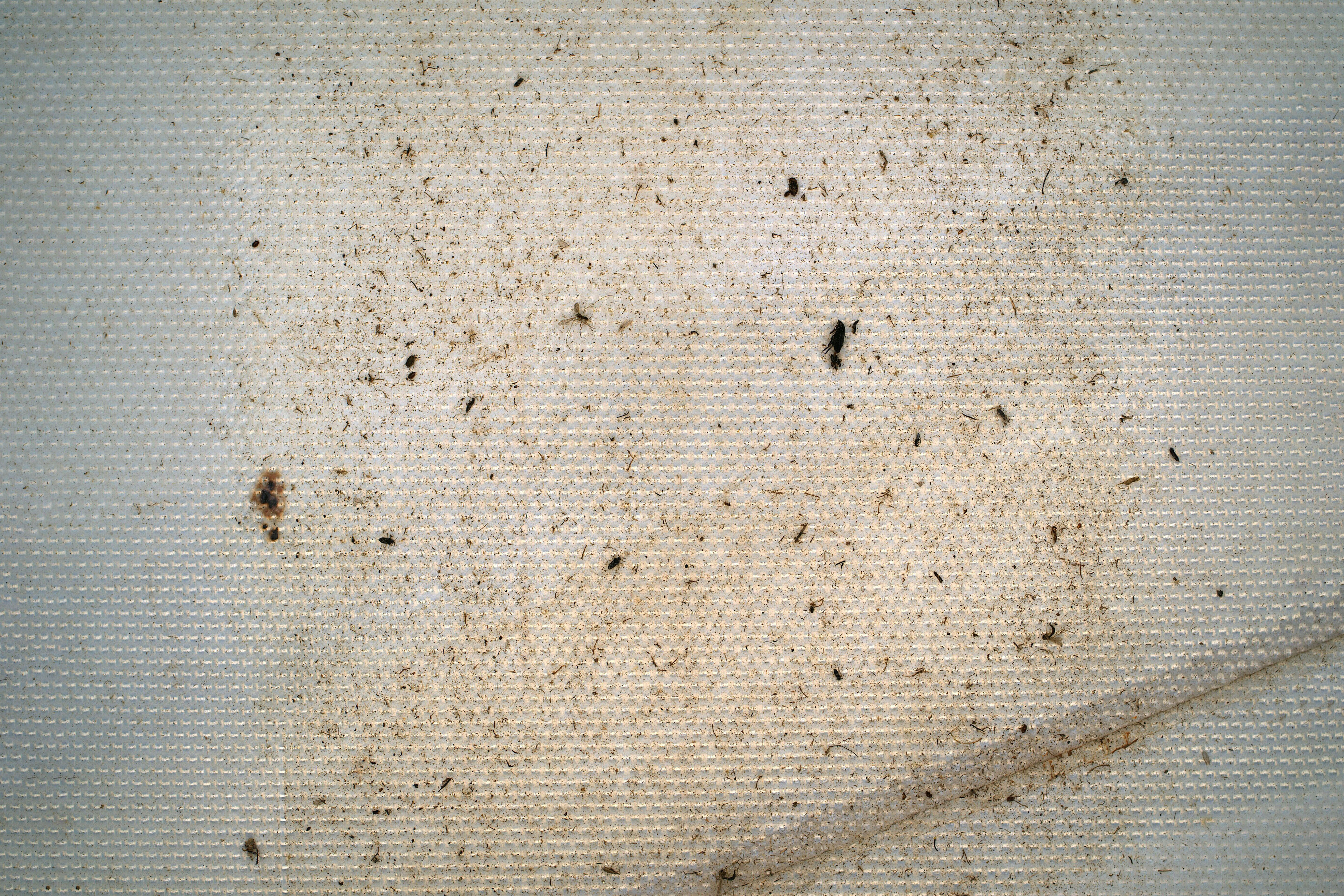

Supplement: S1 Fig — (ZIP) [file pone.0304284.s001.zip › 0013.jpg]

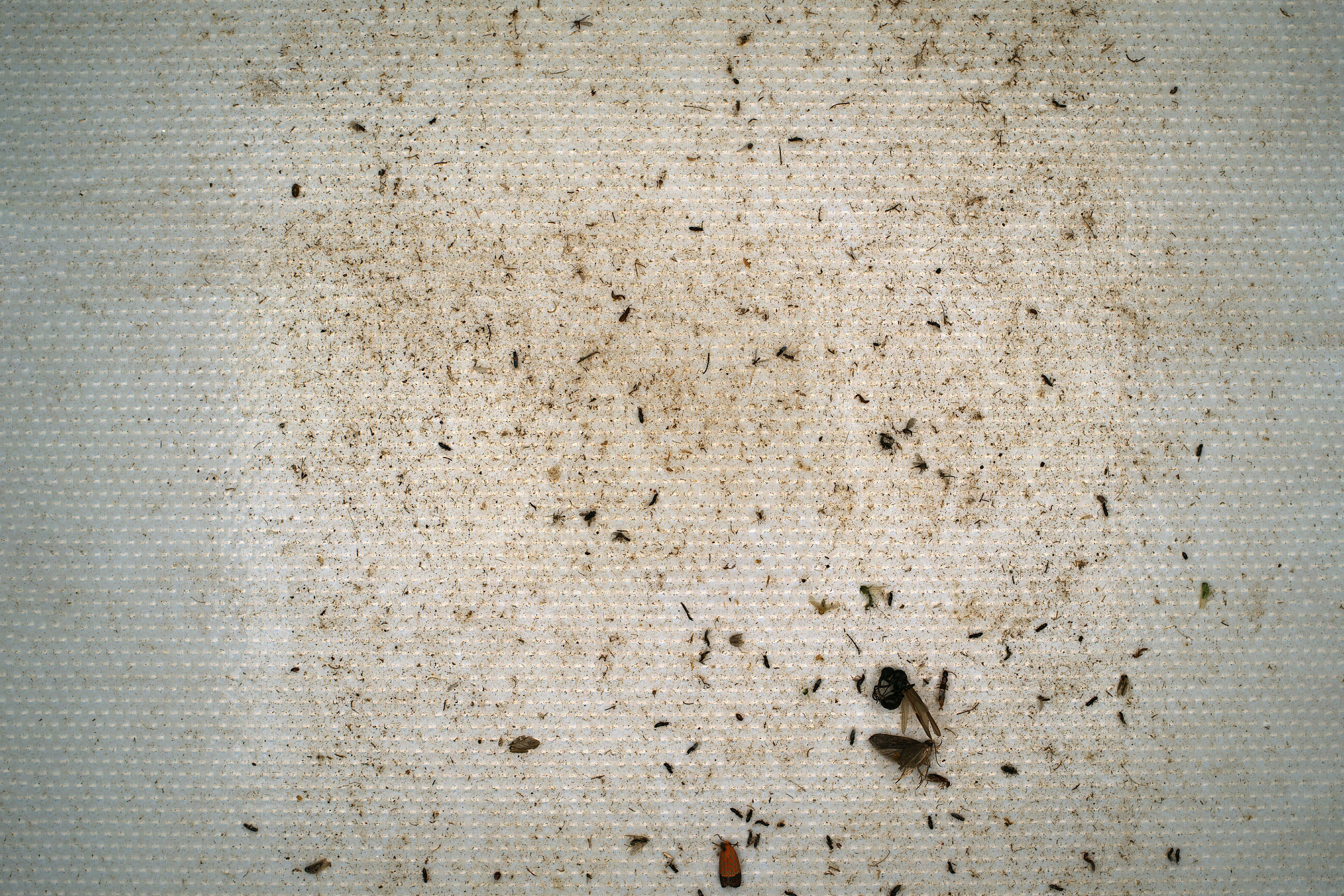

Supplement: S1 Fig — (ZIP) [file pone.0304284.s001.zip › 0014.jpg]

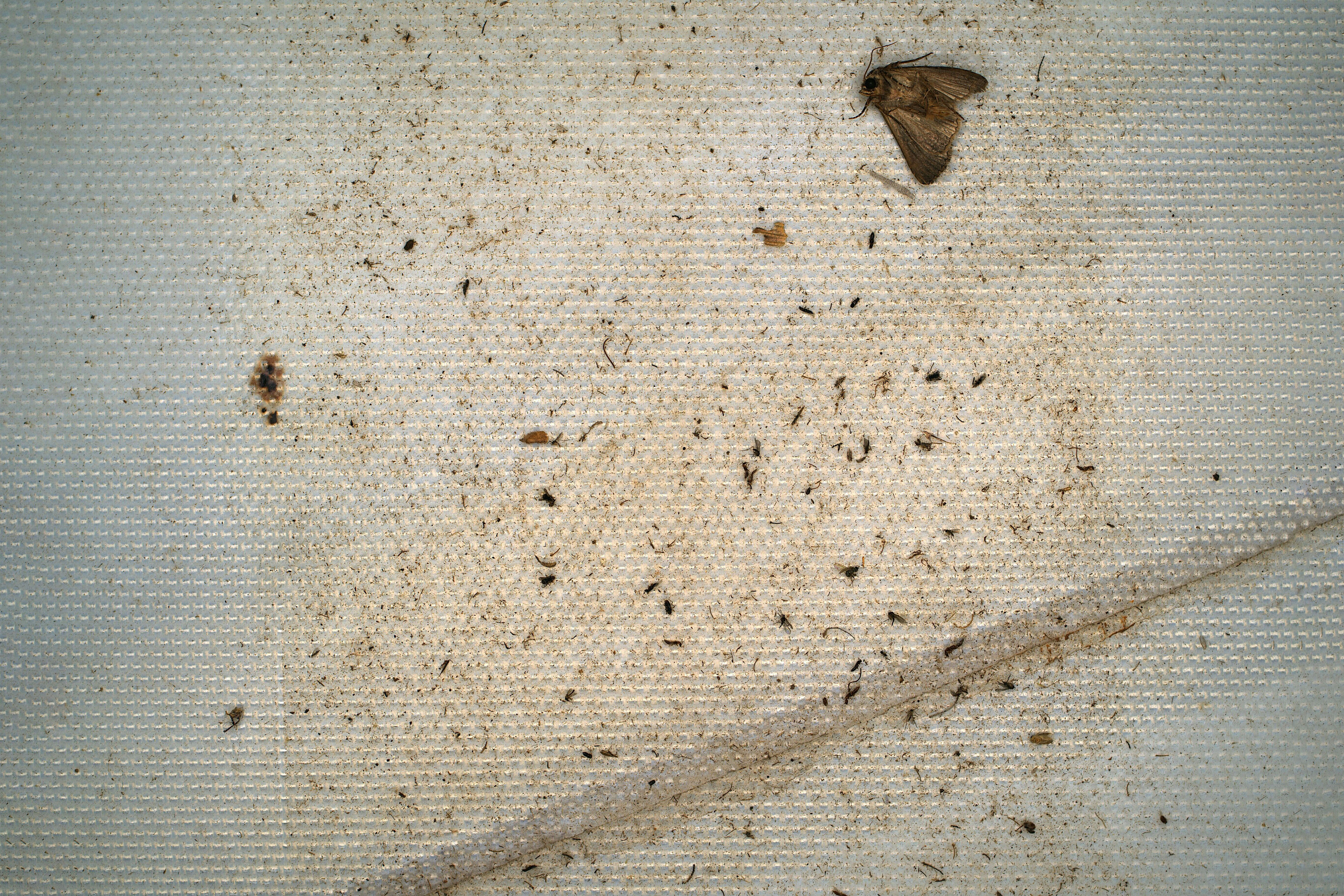

Supplement: S1 Fig — (ZIP) [file pone.0304284.s001.zip › 0015.jpg]
